# Self-assembled templated pulp of *Citrus limetta* porous Biochar@H_3_PO_4_ activated cathode for zinc-ion hybrid supercapacitors

**DOI:** 10.1016/j.isci.2025.113379

**Published:** 2025-08-16

**Authors:** Manisha Gautam, Tarun Patodia, Rahul Vaish, Kanupriya Sachdev, Himmat S. Kushwah

**Affiliations:** 1 Materials Research Centre, Malaviya National Institute of Technology, Jaipur, Rajasthan, India; 2 Shodh Lab, Suresh Gyan Vihar University, Jaipur, Rajasthan, India; 3 School of Mechanical and Materials Engineering, Indian Institute of Technology, Mandi, Himachal Pradesh, India; 4 Department of Physics, Malaviya National Institute of Technology, Jaipur, Rajasthan, India; 5 Department of Physics, Manipal University Jaipur, Jaipur, Rajasthan, India

**Keywords:** Applied sciences, Chemistry, Electrochemical energy storage

## Abstract

This study investigates the utilization of *Citrus limetta* waste pulp as a renewable agro-waste-derived material for energy storage applications, specifically in zinc-ion hybrid supercapacitors (ZIHSCs). Soft-templated synthesis is employed to synthesize the porous template biochar (CL@CTAB_850°C) through hydrothermal treatment followed by pyrolysis at 850°C. To boost the surface properties of the porous template, it undergoes further chemical treatment (H_3_PO_4_), resulting in improved physicochemical and electrochemical performance. The fabricated ZIHSC device exhibits high electrochemical performance for CL@CTAB@H_3_PO_4__850°C cathode. The scanning electron microscopy (SEM) and Brunauer-Emmett-Teller (BET) results confirm the porous nature of the materials. The ZIHSC device exhibits excellent self-discharge behavior with high voltage retention, addressing key challenges in current energy storage technology and advancing its potential for practical implementations. This research highlights a resource-efficient and waste-valorizing approach for creating high-performance carbon electrodes, demonstrating the potential of agro-waste valorization in energy storage and ZIHSC devices.

## Highlights

• *Citrus limetta* (CL) pulp serves as a biomass raw material
• This research compares and analyses zinc-ion hybrid supercapacitor (ZIHSC) devices
• The porous carbon template was created using CTAB as a soft-templating agent
• Dual activation of *Citrus limetta*-derived biochar boosts the electrochemical performance

## Graphical abstract

**Figure:**
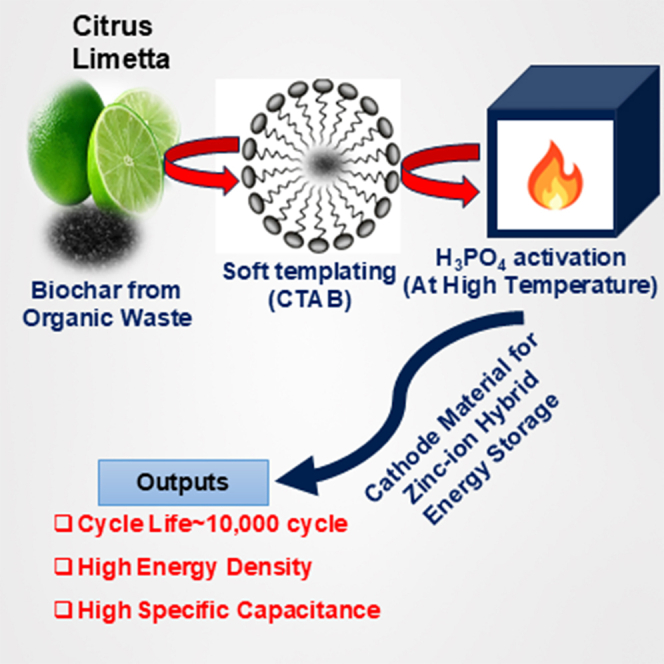

## Introduction

Energy storage technologies are essential for meeting the growing demands of electric vehicles, sensors, and portable devices.^1^ Lithium-ion batteries, first commercialized in 1991, have received significant interest owing to their high energy density and extended lifespan. However, issues include safety risks, low power output, limited cycle life, rising cost, and the scarcity of lithium resources.^2^^,^^3^ In contrast, the electric double layer offers excellent power delivery and fast charging rates but suffers from low energy density.^4^ This has driven the research toward hybrid supercapacitor systems that combine the key features of batteries and electric double-layer supercapacitors to achieve energy and power densities.^5^^,^^6^

Zinc-ion hybrid supercapacitors (ZIHSCs) have emerged as viable alternatives, offering low cost, environmental compatibility, improved safety, high energy and power performance, and robust cycling stability.^7^ Aqueous ZIHSCs, which assemble a battery-type anode with a capacitor-type cathode, are considered highly favorable due to their ability to integrate the advantages of zinc-ion batteries and supercapacitors in a single device, with the superior energy and power densities. Zinc metal is especially attractive due to its natural abundance and low redox potential (−0.76) vs. the standard hydrogen electrode.^8^ Despite these benefits, ZIHSCs experience difficulties in limited energy density and kinetic mismatch between electrodes, significantly hindering the commercial development of ZIHSCs.^9^ Consequently, designing and optimizing cathode materials is crucial to advancing ZIHSCs toward practical and large-scale applications.^10^ Activated carbon, porous carbon, and graphene are commonly utilized as carbon-based cathodes in ZIHSCs due to their low cost, chemical stability, tunable structure, and excellent electrical conductivity.^11^^,^^12^ However, conventional synthesis routes often involve concentrated acid and harsh conditions, limiting environmental compatibility and scalability.^13^ Biomass-derived carbon materials have emerged as a promising alternative owing to their renewability and low cost. Biochar synthesized from waste biomass exhibits excellent catalytic and adsorption properties, making it a renewable resource for energy storage applications.^14^ Key physicochemical characteristics, such as adsorption capacity, pore size distribution (PSD), and specific surface area (SSA), are critical in optimizing electrochemical performance.^15^ Utilizing carbon compounds from biomass is a viable approach to energy storage.^16^ Therefore, biomass-derived carbon cathode offers a resource-efficient and practical pathway for advancing high-performance ZIHSCs.^17^

Highly ordered mesoporous carbons derived from organic sources such as forestry and agricultural wastes offer a versatile approach for energy storage applications. These materials exhibit excellent thermal and mechanical stability.^18^ Soft templating, which utilizes surfactant micelles, is a promising approach for synthesizing mesoporous carbon materials with well-defined pore structures, although it typically yields smaller pore sizes and lower surface areas.^19^ Additional chemical or physical modification is often necessary to enhance surface area, porosity, and functional groups.^20^ Activation enhances the ion transport and charge storage efficiency by introducing micropores (≤2 nm), mesopores (2–50 nm), and macropores (>50 nm).^21^ Chemical activation modifies biomass-derived carbons’ pore structure and surface functionality, indirectly modulating electric double-layer capacitance (EDLC) performance. This modulation arises from micro- and mesoporous architecture changes, which govern ion transport dynamics, double-layer formation, and overall capacitance behavior.^22^^,^^23^ For instance, Zhou et al. developed a flexible ZIHSC using cellulose nanofibers hydrogel and porous carbon from oil palm wood, achieving an energy density of 53.7 Wh Kg^−1^.^24^ Zhimin et al. utilized corn silk-derived mesoporous carbons with high SSA and pore volume, resulting in an energy density of 25 Wh Kg^−1^ at a power density of 23.07 kW kg^−1^ and showed 87.5% capacitance retention after 10,000 cycles for ZIHSCs.^25^ Zhang et al. developed nitrogen (N) and oxygen (O) co-doped carbon micro foam using gelatin as precursor through pre-carbonization, followed by KOH activation with an enormous surface area (greater than 3,000 m^2^ g^−1^) in organic and aqueous electrolytes, and showed remarkable energy density (90 Wh Kg^−1^) for ZIHSCs.^26^ Yao et al. utilized sweet mess from the alcoholic fermentation of glutinous rice to develop glutinous rice porous carbon (GRPC-A13), a carbon-based electrode for ZIHSC with a high energy density of 116 Wh Kg^−1^ (at 800 W kg^−1^) and 100% capacitance retention at 5 A g^−1^.^27^ Our previous study used banana peel as a low-cost biomass source for high-performance ZIHSC with H_3_PO_4_ activation, yielding biochar with enhanced surface area and achieved an energy density of 120 Wh Kg^−1^.^28^ These results emphasize the potential of activated carbon derived from chemical treatment of biomass/biochar for energy storage applications. Based on previous encouraging findings, the present study aims to use a soft-templating approach to investigate alternative biomass feedstocks for porous carbon synthesis. Despite the abundance of biomass-based research, there remains a significant lack of high-performance cathode materials for ZIHSCs. Based on the available literature, no studies have reported on the valorization of *Citrus limetta* biomass in this context, highlighting the novelty of the present work. *Citrus limetta*, a widely consumed fruit, generates considerable biomass residues during the processing. Its pulp is rich in bioactive compounds such as vitamin C, folic acid, and phenolics, contributing to its nutritional value and chemical significance.^23^

This study presents a novel synthesis of mesoporous biochar derived from *Citrus limetta* pulp using cetyltrimethylammonium bromide (CTAB) as a soft-template agent, followed by chemical activation with phosphoric acid (H_3_PO_4_). The resulting activated biochar derived from the pulp of *Citrus limetta* exhibits a high surface area and porous structure, which can facilitate electric double-layer charge storage when used as a cathode material in ZIHSC applications. The optimized electrode (CL@CTAB@H_3_PO_4__850°C) achieved an outstanding specific capacitance (904 F g^−1^ at 0.1 A g^−1^ current density) and a remarkably high energy density (321.66 Wh Kg^−1^). Furthermore, both the soft-templating-derived electrode (CL@CTAB_850°C) and the H_3_PO_4_ activated electrode (CL@H_3_PO_4__850°C) demonstrated the exceptional Coulombic efficiencies for 10,000 galvanostatic charge-discharge (GCD) cycles. The feasibility underscores the viability of agro-waste sources and soft-templating approaches in evolving cost-effective and high-performance cathodes for energy storage applications.

## Results and discussion

### Material analysis

**Figure 1:**
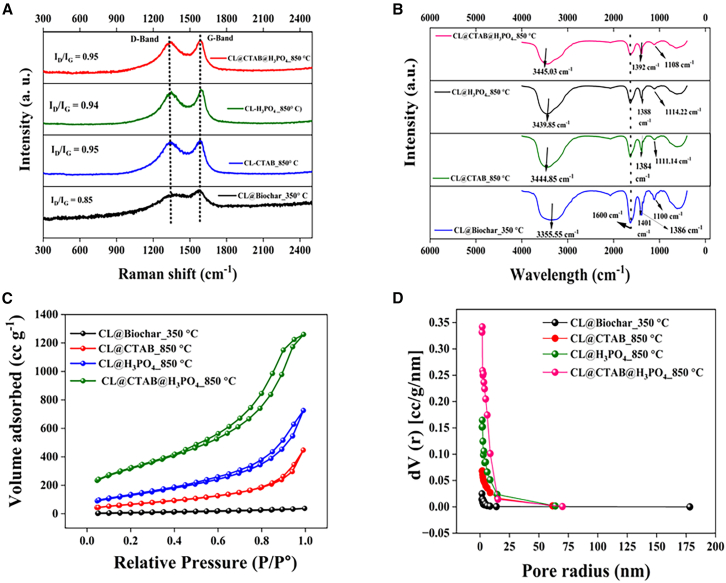
Material characterization analysis for CL@Biochar_350°C, CL@CTAB_850°C, CL@H_3_PO_4__850°C, and CL@CTAB@H_3_PO_4__850°C (A) Raman analysis, (B) FTIR analysis, (C) BET isotherm, and (D) pore size distribution.

**Figure 2:**
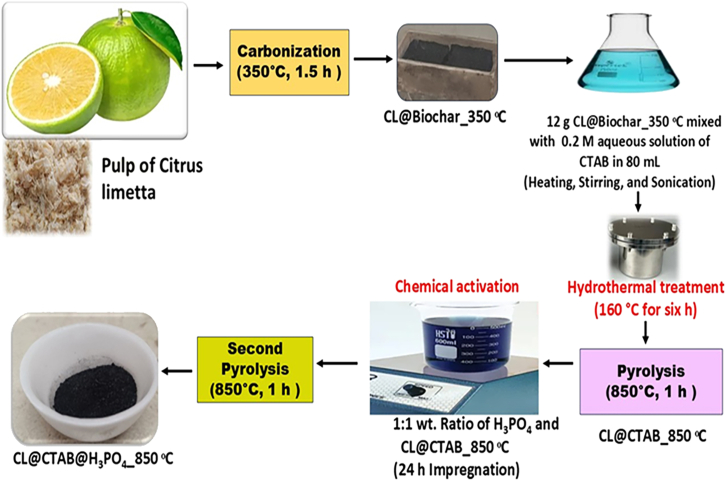
The synthesis process of porous carbon was done using the CTAB surfactant and further treated with the H_3_PO_4_ activation method

Figure 1 presents the Raman spectra (Figure 1A), Fourier transform infrared (FTIR) spectra (Figure 1B), BET isotherm (Figure 1C), and pore structure analysis curves (Figure 1D) for the synthesized carbon materials (as shown in Figure 2): CL@Biochar_350°C, CL@CTAB_850°C, CL@H_3_PO_4__850°C, and CL@CTAB@H_3_PO_4__850°C activated carbons.

In the Raman spectra (Figure 1A), all sample exhibits two prominent peaks observed near the G-band (∼1,580 cm^−1^) and the D-band (∼1,350 cm^−1^), which are characteristic of carbon materials. The D-band arises due to structural disorder and defects, whereas the G-band arises from the in-plane vibration of sp^2^ hybridized carbon.^24^ The intensity ratio (I_D_/I_G_) increases after soft templating and chemical activation, indicating the introduction of defects into the carbon framework due to high-temperature treatment and phosphoric acid activation. In contrast, pristine CL@Biochar_350°C exhibits a lower D-band intensity, suggesting a more ordered carbon structure with lower defect density.

Figure 1B displays the FTIR spectra, revealing characteristic absorption bands associated with different functional groups. All samples exhibit a broad adsorption band around 3,400 cm^−1^, attributed to O-H stretching vibrations from hydroxyl groups and adsorbed water. A distinct peak near 1,630 cm^−1^ arises from the C=C stretching vibrations of aromatic carbon structures. Peaks appearing in the 1,300–1,400 cm^−1^ region are associated with C-H bending and P-O stretching vibration, confirming the successful incorporation of phosphate groups due to H_3_PO_4_ activation. A weak band at approximately 2,069 cm^−1^ is also attributed to C-H stretching vibration from aliphatic hydrocarbon species. For the CL@CTAB_850°C, the peak at 1,401 cm^−1^ is associated with the C-H bending vibration, and the peak at 1,111.4 cm^−1^ corresponds to vibrations of C-O stretching. For CL@H_3_PO_4__850°C, the peak at 1,108 cm^−1^ is present; phosphate or polyphosphate groups are causing C-O-P stretching vibrations. For CL@CTAB@H_3_PO_4__850°C, the peak at 1,114.2 cm^−1^ corresponds to phosphate or polyphosphate groups, which cause C-O-P stretching vibrations. The slight shift observed from 1,108 cm^−1^ (CL@H_3_PO_4__850°C) to 1,114.2 cm^−1^ (CL@CTAB@H_3_PO_4__850°C) may be attributed to changes in the phosphate bonding environment caused by CTAB pre-treatment.^29^^,^^30^^,^^31^

Figure 1C presents the BET isotherm analysis. The isotherm exhibits the features of both type I and IV hysteresis loops, indicating the presence of micro- and mesoporous structure due to monolayer and multilayer adsorption. In porous materials, pore condensation phenomena occur at higher relative pressures, and the volume of the gas adsorbed determines the surface area. The CL@Biochar_350°C sample exhibits minimal adsorption, with a low surface area of 37.24 m^2^ g^−1^, indicating limited porosity. In contrast, CL@CTAB_850°C and CL@H_3_PO_4__850°C exhibit significantly increased surface areas of 256.68 and 486.2 m^2^ g^−1^, respectively, attributed to soft templating and chemical activation. The highest BET surface area was achieved by CL@CTAB@H_3_PO_4__850°C, reaching 1,132.81 m^2^ g^−1^, highlighting the synergetic effect of dual activation. The PSD analysis of *Citrus limetta*-derived samples revealed porous features across all the samples, with pore sizes ranging from 1.08 to 1.91 nm and corresponding pore volume increasing significantly with surface modification, as shown in Figure 1D and Table 1. Interestingly, the dual-modified sample (CL@CTAB@H_3_PO_4__850°C) showed a slightly reduced pore size of 1.68 nm but maintained a high pore volume of 1.6 cc g^−1^, indicating the development of highly accessible and interconnected porous networks.

**Table 1:** Comparison of Brunauer-Emmet-Teller (BET) surface area and pore size distribution analysis of *Citrus limetta*-derived activated biochar materials

*Citrus limetta*	Surface area (m^2^ g^−1^)	Pore size (nm)	Pore volume (cc g^−1^)
CL@Biochar_350°C	37.24	1.70	0.055
CL@CTAB_850°C	256.68	1.90	0.65
CL@H_3_PO_4__850°C	480.32	1.91	1.7
CL@CTAB@H_3_PO_4__850°C	1,132.80	1.68	1.6

**Figure 3:**
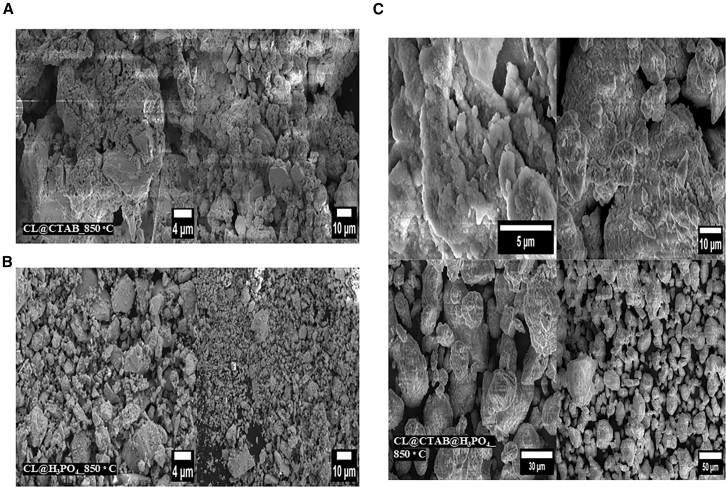
Field-emission scanning electron microscopy analysis of citrus limetta-derived materials (A) CL@CTAB_850°C, (B) CL@H_3_PO_4__850°C, and (C) CL@CTAB@H_3_PO_4__850°C.

Figure 3 displays field-emission scanning electron microscopy (FE-SEM) images of the carbon material derived from the different synthesis routes: CL@CTAB_850°C, CL@H_3_PO_4__850°C, and CL@CTAB@H_3_PO_4__850°C at various magnifications. Figure 3A shows the FE-SEM images of CL@CTAB_850°C at 4 and 10 μm scales. The material exhibits a highly fragmented, rough, and irregular surface topology. The morphology indicates partly broken carbon flakes and loosely aggregated microstructure, suggesting that CTAB-assisted soft templating leads to moderated porosity and structure disruption during carbonization at 850°C. This roughness is indicative of micropore formation. Figure 3B corresponds to the FE-SEM images of CL@H_3_PO_4__850°C at the same magnifications. The surface is densely packed with irregularly shaped particles, displaying significantly more fragmentation and finer micro-grain features than the CTAB-treated sample. This morphology results from phosphoric acid activation, which induces extensive pore formation through chemical etching and dehydration reaction during carbonization. The observed fine particles suggest enhanced surface area and micro- and mesopores. Figure 3C illustrates the morphological features of CL@CTAB@H_3_PO_4__850°C at 5, 10, 30, and 50 μm magnifications. This sample demonstrates the most well-developed porosity and structure morphology among all. The surface has a spherical, wrinkled structure and highly porous, sponge-like textures. The co-treatment with CTAB and H_3_PO_4_ facilitated the formation of hierarchical pores and enhanced surface roughness and porosity. Numerous uniformly dispersed spherical carbon granules are visible at higher magnifications (30–50 μm). This suggests a successful templating and activation strategy, explaining the observed highest SSA in the BET analysis.

### Electrochemical analysis

**Figure 4:**
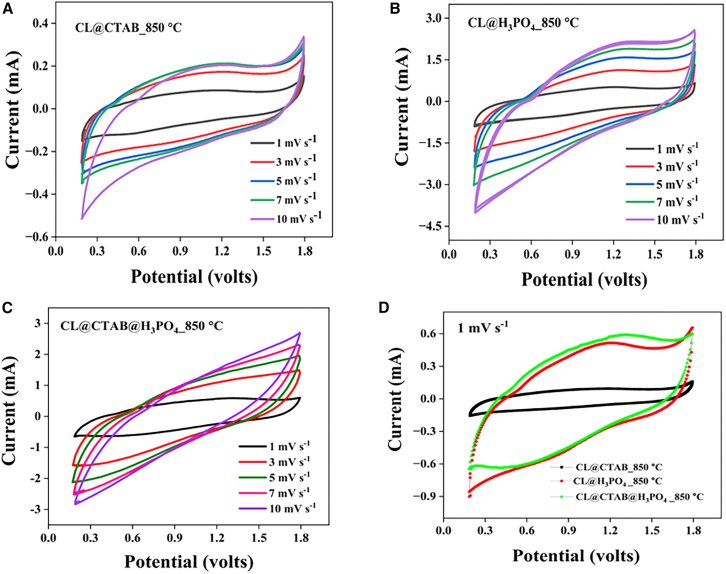
Cyclic voltammetry analysis for ZIHSC devices (A) Zn//2M ZnSO_4_//CL@CTAB_850°C, (B) Zn//2M ZnSO_4_//CL@H_3_PO_4__850°C, and (C) Zn//2M ZnSO_4_//CL@CTAB@H_3_PO_4__850°C ZIHSC devices and (D) comparative analysis of CV curves at 1 mV s^−1^ scan rate.

**Figure 5:**
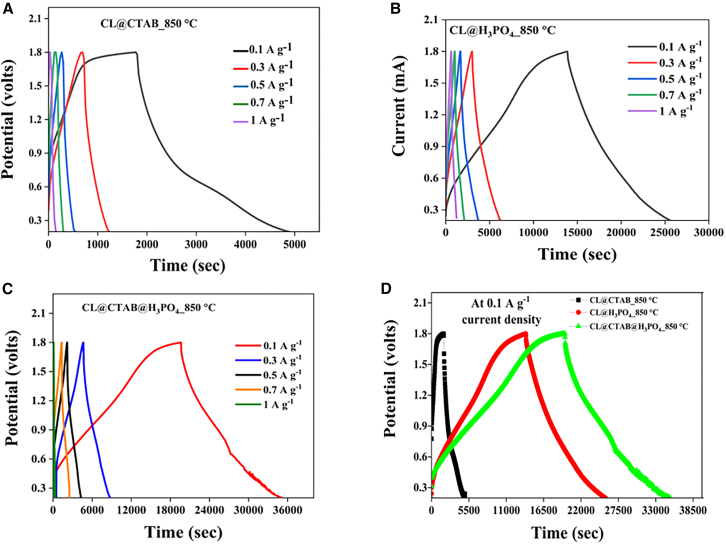
GCD analysis for ZIHSC devices (A) Zn//2M ZnSO_4_//CL@CTAB_850°C, (B) Zn//2M ZnSO_4_//CL@H_3_PO_4__850°C, and (C) Zn//2M ZnSO_4_//CL@CTAB@H_3_PO_4__850°C ZIHSC devices and (D) comparison analysis of GCD curves at the current density of 0.1 A g^−1^.

The electrochemical study of the cathode materials was systematically evaluated using cyclic voltammetry (CV) and GCD analysis, as illustrated in Figures 4 and 5. Specifically, Figure 4 presents the CV profiles of the ZIHSC devices: Zn//2M ZnSO_4_//CL@CTAB_850°C, Zn//2M ZnSO_4_//CL@H_3_PO_4__850°C, and Zn//2M ZnSO_4_//CL@CTAB@H_3_PO_4__850°C.

Figure 4A presents the CV curves of the Zn//2M ZnSO_4_//CL@CTAB_850°C device at various scan rates ranging from 1 to 100 mV s^−1.^ The CV curves exhibit semi-rectangular shapes with merely symmetric oxidation and reduction behavior, suggesting a combination of electrochemical double-layer capacitance and pseudo-capacitance. The peak current values increase with increasing scan rates, indicating excellent electrochemical reversibility and rate capability. Similar behavior was observed for the Zn//2M ZnSO_4_//CL@H_3_PO_4__850°C device as shown in Figure 4B and for the Zn//2M ZnSO_4_//CL@CTAB@H_3_PO_4__850°C device as shown in Figure 4C. The comparative CV analysis at a low scan rate of 1 mV s^−1^, shown in Figure 4D, revealed that the Zn//2M ZnSO_4_//CL@CTAB@CL@H_3_PO_4__850°C device exhibits the largest electrochemical surface area. This enhancement is due to dual activation effects, increasing surface heterogeneity.

The CV data confirm that all three ZIHSC configurations exhibit ideal capacitive behavior. This is consistent with Randle Sevik’s equation (Equation 1), which corresponds to the peak current (i_p_) being linearly dependent on the scan rate (ϑ),

(Equation 1) $$ip=2.69\times 10^{5\;}\times n^{\frac{3}{2\;}}\times A\times C\times \sqrt{D\vartheta }$$

where n = number of electrons transferred due to redox reactions

A = area of electrode (cm^2^)
C = concentration (mol/cm^3^)
ϑ = scan rate (V/S).

To elucidate the charge storage mechanism, Dunn’s model (Equation 2) was employed, which differentiated the surface-controlled capacitive and diffusion-controlled processes based on the power law relationship between peak current and scan rate:^32^^,^^33^

(Equation 2) $$i=a\vartheta^{b}$$

By taking logs on both sides, this equation can be written in different forms,

(Equation 3) Log(i)=bLog(ϑ)+Log(a)

Here, the b value provides insight into the charge storage mechanism, which can be calculated by plotting the graph between log (i) against log (ϑ)

(1) b = 0.5 (diffusion-controlled process)
(2) b = 1 (capacitive control process)
(3) 0.5 < b < 1 suggests the mixed behavior

The computed b values are approximately 0.58 for Zn//2 M ZnSO_4_//CL@CTAB_850°C, 0.6 for Zn//2M ZnSO_4_//CL@H_3_PO_4__850°C, and 0.3 for Zn//2M ZnSO_4_//CL@H_3_PO_4__850°C as shown in Figure S1A, suggesting a dominant diffusion-controlled mechanism at lower scan rates.

Additionally, the contributions of capacitive and diffusion-controlled processes were quantitatively assessed through analysis of current response at various scan rates using Equations 4 and 5:

(Equation 4) $$I\;\left(V\right)=k_{1}\vartheta +k_{2}\vartheta^{0.5}$$

(Equation 5) $$\frac{I\;\left(V\right)}{\vartheta^{0.5}}=k_{1\;}V+k_{2}$$

By plotting I (V)/ϑ^0.5^ and ϑ^0.5^, the relative contributions of capacitive (k_1_ϑ) and diffusion-controlled (k_1_ϑ^0.5^) currents were examined. The Zn//2M ZnSO_4_//CL@CTAB_850°C device exhibited the dominant diffusion-controlled behavior at low scan rates with diffusion contribution decreasing from 98% at 1 mV s^−1^ to 30% at 100 mV s^−1^, as illustrated in Figure S1B. In contrast, the Zn//2M ZnSO_4_//CL@H_3_PO_4__850°C device achieved consistently high capacitive contribution, reaching 100% at scan rates of ≥50 mV s^−1^ as demonstrated in Figure S1C. The Zn//2M ZnSO_4_//CL@CTAB@H_3_PO_4__850°C device displayed a hybrid behavior, with the diffusion and capacitive contributions gradually shifting from 89% and 11% (at 1 mV s^−1^) to 45% and 55% (at 100 mV s^−1^), respectively, as illustrated in Figure S1D. The Zn//CL@H_3_PO_4_ device showed a strong capacitive dominance across all scan rates, reaching 100% capacitive behavior at 50 mV s^−1^ and above, with the diffusion control ratio dropping nearly to zero. In contrast, the Zn//2M ZnSO_4_//CL@CTAB@H_3_PO_4__850°C device exhibited mixed contributions. This suggests that a hybrid electrode structure enhances both EDLC and pseudocapacitive mechanisms.

Overall, the b values and charge storage contributions confirm the following.

(1) At lower scan rates, capacitive is predominantly governed by faradaic or diffusion-controlled reaction.
(2) At higher scan rates, surface-controlled process (EDLC) becomes more prominent.
(3) The Zn//CL@CTAB@H_3_PO_4__850°C device demonstrates a unique hybrid energy storage behavior, befitting of both EDLC and pseudo-capacitance due to its optimized structure and activation strategy.

The electrochemical performance of ZIHSC devices was further evaluated through GCD analysis, as shown in Figure 5. The GCD profiles for Zn//2M ZnSO_4_//CL@CTAB_850°C, Zn//2M ZnSO_4_//CL@H_3_PO_4__850°C, and Zn//2M ZnSO_4_//CL@CTAB@H_3_PO_4__850°C devices were systematically studied. Figure 5A shows the GCD curves of the Zn//2M ZnSO_4_//CL@CTAB_850°C device at various current densities from 0.1 to 1 A g^−1^. The GCD profile exhibits noticeable non-linearity, indicating a combination of EDLC and pseudocapacitive behavior. The curves also display good symmetry between charge and discharge plots, suggesting excellent electrochemical reversibility. As the current density increases, the charge/discharge times decrease due to limited ion diffusion and shortened time available for ion intercalation at higher scan rates. Similar behavior was observed for Zn//2M Zn//2M ZnSO_4_//CL@H_3_PO_4__850°C as shown in Figure 5B and the Zn//2M ZnSO_4_//CL@CTAB@H_3_PO_4__850°C device as shown in Figure 5C. At lower current densities, prolonged charge-discharge durations were observed, attributed to slower ion diffusion and enhanced interaction between electrolyte ions and electrode surface. In contrast, higher current densities promoted faster ion kinetics, albeit with reduced charge/discharge tolerance due to insufficient ion diffusion and limited access to inner active sites. Figure 5D presents a comparative analysis of the GCD curve for Zn//2M ZnSO_4_//CL@CTAB_850°C, Zn//2M ZnSO_4_//CL@H_3_PO_4__850°C, and Zn//2M ZnSO_4_//CL@CTAB@H_3_PO_4__850°C devices at a low current density. Among them, the Zn//2M ZnSO_4_//CL@CTAB@H_3_PO_4__850°C device demonstrated the longest charge-discharge duration, indicative of higher charge storage capacity.

Furthermore, the specific capacities of the ZIHSC devices were derived from the GCD data and plotted against the voltage, as illustrated in Figure S2. Figures S2A–S2C show the specific capacity profiles at varying current densities (0.1 to 1 A g^−1^) for Zn//2M ZnSO_4_//CL@CTAB_850°C (Figure S2A), Zn//2M ZnSO_4_//CL@H_3_PO_4__850°C (Figure S2B), and Zn//2M ZnSO_4_//CL@CTAB@H_3_PO_4__850°C (Figure S2C) devices, respectively. All devices exhibited excellent reliability and consistent performance across different current densities. Figure S2D presents a comparative analysis of the specific capacity at low current density. The Zn//2M ZnSO_4_//CL@CTAB@H_3_PO_4__850°C device delivered the highest specific capacity, further validating the benefit of dual activation in enhancing electrochemical performance. These findings are consistent with CV and GCD analysis, highlighting the supercapacitor charge storage characteristic of the CL@CATB@H_3_PO_4__850°C cathode configuration.

**Figure 6:**
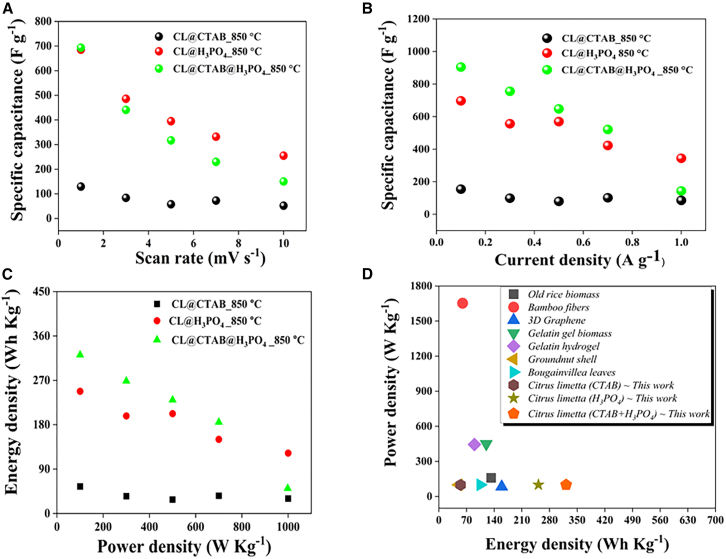
Comparative analysis of ZIHSC devices’ parameters (A) Specific capacitance vs. scan rate; (B) specific capacitance vs. current density; and (C) energy density vs. power density relationship for Zn//2M ZnSO_4_//CL@CTAB_850°C, Zn//2M ZnSO_4_//CL@H_3_PO_4__850°C, and Zn//2M ZnSO_4_//CL@CTAB@H_3_PO_4__850°C ZIHSC devices and (D) Ragone plot for all ZIHSC devices.

As shown in Figure 6, the specific capacitance values of the fabricated ZIHSCs were evaluated under various scan rates and current densities. These values were calculated using Equations 6 and 7,^34^^,^^35^derived from CV and GCD measurements.

(Equation 6) $$S.C\;\left(F\;g^{-1}\right)=\text{From}\;\text{CV}\;\frac{Area\;of\;CV\;curve}{Scan\;rate\left({mV\;S}^{-1}\right)\times mass\;of\;active\;material\;\left({mg\;cm}^{-2}\right)\times Voltage\;window\;\left(volts\right)}$$

From the GCD analysis based on the non-linear charge-discharge curves

(Equation 7) $$S.C\;\left(F\;g^{-1}\right)=\frac{2\times I\times A}{m\times {\Delta V}^{2}}$$

where

I = current density (A g^−1^)
A = area (under the discharge curve)
m = mass loading (mg cm^−2^)
ΔV = potential window (volts)

Furthermore, energy and power densities were calculated using Equations 8 and 9,^33^^,^^34^ respectively. Additionally, the relationship between energy density and power densities at various current densities was shown in Figure 6.

Equations 8 and 9 were defined as

(Equation 8) $$Energy\;density\;\left(Wh\;{Kg}^{-1\;}\right)=\frac{Specific\;capacitance\times {\Delta V}^{2}}{7.2}$$

(Equation 9) $$Power\;density\;\left(Wh\;{Kg}^{-1}\right)=\frac{3600\times Energy\;density\;}{Discharge\;time\;\left(\Delta t\right)}$$

Among the devices tested, Zn//2M ZnSO_4_//CL@CTAB@H_3_PO_4__850°C demonstrated the highest specific capacitance and electrochemical performance. When compared with Zn//2M ZnSO_4_//CL@H_3_PO_4__850°C and Zn//2M ZnSO_4_//CL@CTAB_850°C, the cathode material synthesized using both CTAB soft templating and phosphoric acid activation showed superior performance due to the synergetic effects of the dual-modification approach. The specific capacitance values at scan rates of 1 and 10 mV s^−1^ for Zn//2M ZnSO_4_//CL@CTAB_850°C were 129.51 and 51.87 F g^−1^, respectively. For Zn//2M ZnSO_4_//CL@H_3_PO_4__850°C, the values are 685.08 and 255 F g^−1^, and, for Zn//2M ZnSO_4_//CL@CTAB@H_3_PO_4__850°C, the values are 693.50 and 150 F g^−1^, respectively, as shown in Figure 6A. Table S1 incorporates the specific capacitance value at the scan rates of 1, 3, 5, 7, and 10 mV s^−1^ for all ZIHSC devices. The Zn//2M ZnSO_4_//CL@CTAB@H_3_PO_4__850°C device also showed the highest specific capacitance, particularly at a lower current density of 0.1 A g^−1^, confirming the effectiveness of the synthesis strategy. At current densities of 0.1, 0.3, 0.5, 0.7, and 0.1 A g^−1^, the specific capacitance values obtained for Zn//2M ZnSO_4_//CL@CTAB_850°C are 154.74, 98.98, 79.77, 101.66, and 85.56 F g^−1^, respectively. The corresponding values for Zn//2M ZnSO_4_//CL@H_3_PO_4__850°C were 697, 556.44, 569.45, 422.82, and 345 F g^−1^, respectively. The Zn//2M ZnSO_4_//CL@CTAB@H_3_PO_4__850°C device outperforms both, obtaining specific capacitance values as 904.6, 755, 647.82, 521.64, and 143.7 F g^−1^, respectively, as shown in Figure 6B and Table S2. The enhanced electrochemical performance of Zn//2M ZnSO_4_//CL@CTAB@H_3_PO_4__850°C can be attributed to the increased porosity and high surface area achieved through the combined activation and the soft-templating approach. The rate performance of the ZIHSC devices determined by the GCD measurements indicates good rate capability. The Zn//2M ZnSO_4_//CL@CTAB_850°C device achieved 64.9%, while the Zn//2M ZnSO_4_//CL@H_3_PO_4_ device exhibited the 60.54% and the Zn//2M ZnSO_4_//CL@CTAB@H_3_PO_4__850°C device achieved the 58% of rate performance. These results confirm the excellent electrochemical behavior of the devices and effective electrolyte ion transport even at higher current densities.

Figure 6C shows the relationship between the energy and power densities at various current densities (ranging from 0.1 to 1 A g^−1^) of the fabricated ZIHSC devices. Table S3 incorporates the energy and power density values for the Zn//2M ZnSO_4_//CL@CTAB_850°C, Zn//2M ZnSO_4_//CL@H_3_PO_4__850 ͦ C, and CL@CTAB@H_3_PO_4__850°C ZIHSC devices. Among all the ZIHSC devices, the Zn//2M ZnSO_4_//CL@CTAB@H_3_PO_4__850°C device delivers the highest energy density (321.6 Wh Kg^−1^) at 0.1 A g^−1^ current density, outperforming both the Zn//2M ZnSO_4_//CL@CTAB_850°C (55.02 Wh Kg^−1^) and Zn//2M ZnSO_4_//CL@H_3_PO_4__850°C (247.82 Wh Kg^−1^) devices. However, the energy density decreases with the increasing current density of all the devices. All three ZIHSC devices exhibit a linear increase in power density with current density, achieving up to 1,000 W kg^−1^ power density at 1 A g^−1^. This performance suggests that CL@CTAB@H_3_PO_4__850°C cathode materials are most suitable for applications requiring both energy and power performance.

Figure 6D presents the Ragone plot, highlighting the performance of current devices alongside literature-reported ZIHSC based on biomass-derived cathodes. In comparison, Song et al. reported porous carbon derived from the carbonization of puffed rice, achieving the SSA of 1,651.85 m^2^ g^−1^, respectively, with enhanced Zn^2+^ adsorption via. nitrogen atom doping and the fabricated ZIHSC have shown a specific capacity of 164.0 mAh g^−1^.^36^ Zheng et al. synthesized phosphorus-doped porous carbon, a potential electrode material for ZIHSCs, using bamboo fibers and phytic acid, achieving the SSA of 1,229 m^2^ g^−1^, specific capacity of 109 mAh g^−1^, and maximum energy density of 60 Wh Kg^−1^ at power density of 1,653 W kg^−1^.^37^ Gauri Sankar Das and colleagues fabricated 3D graphene aerogels from biomass for ZIHSCs as cathode materials and showed an outstanding rate capability up to 10,000 cycles at 10 A g^−1^, as well as high specific capacitance (353.1 F g^−1^ at 0.1 A g^−1^) and maximum specific energy (158.9 Wh Kg^−1^ at 84 W kg^−1^).^38^ Samage et al. converted *Solanum melongena* into activated carbon material for symmetric supercapacitors and ZIHSCs, which showed a high specific capacitance (313.08 F g^−1^), energy density (141.35 Wh Kg^−1^), and power density (6.93 kW kg^−1^).^39^ *Xin Hou* et al. synthesized porous carbon with a hierarchical structure using gelatin and ZnCl_2_ via a one-step carbonization activation approach, achieving a high specific capacitance (337.6 F g^−1^) in 6 M KOH and showed an excellent cycling performance and a high energy density (120 Wh Kg^−1^ at 450 W kg^−1^).^40^ Compared with these recent advancements, the present study demonstrates one of the highest specific capacitance and energy density values, as illustrated in Table S4. However, it still indicates relatively low power density due to slower redox kinetics at higher scan rates and current densities.

**Figure 7:**
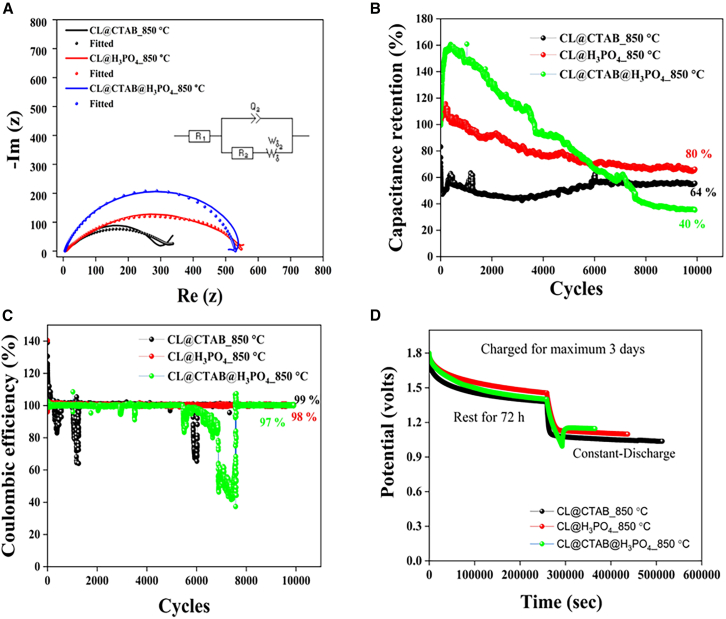
Combined analysis for Zn//2M ZnSO_4_//CL@CTAB_850°C, Zn//2M ZnSO_4_//CL@H_3_PO_4__850°C, and Zn//2M ZnSO_4_//CL@CTAB@H_3_PO_4__850°C ZIHSC devices (A) EIS spectra, (B) capacitance retention, (C) Coulombic efficiency, and (D) self-discharge analysis.

Figure 7 depicts the comparison analysis of ZIHSC devices based on electrochemical impedance spectroscopy (EIS), capacitance retention, Coulombic efficiency, and self-discharge behavior. All fabricated devices underwent EIS analysis over a frequency range of 0.1–100 kHz, as shown in Figure 7A. The EIS measurement revealed two key resistive components: the series resistance (R_s_), which is responsible for electrolyte solution resistance, and the charge transfer resistance (R_ct_), which arises from the formation of the double layer at the electrode-electrolyte interface. The Zn//2M ZnSO_4_//CL@CTAB_850°C device exhibited an R_s_ of 8.26 Ω and an R_ct_ of 430.9 Ω. In comparison, the Zn//2M ZnSO_4_//CL@H_3_PO_4__850°C device showed the lower R_s_ value of 8.09 Ω and higher R_ct_ value of 482.75 Ω. The Zn//2M ZnSO_4_//CL@CTAB@H_3_PO_4__850°C device demonstrated improved impedance characteristics, with an R_s_ value of 5.60 Ω and a lower R_ct_ of 322.9 Ω, indicating efficient ion transport and lower charge transfer resistance due to its optimized porous structure.

To further evaluate the electrochemical durability, we have studied several long-term cycling stability tests for our fabricated ZIHSC devices for 10,000 GCD cycles at a constant current density of 1 A g^−1^, as illustrated in Figure 7B. Among the three devices, the Zn//2M ZnSO_4_//CL@H_3_PO_4__850°C device demonstrates the highest capacitance retention of approximately 80%, confirming the structural advantages imparted by phosphoric acid activation. The stable porous carbon network and enhanced wettability supported stable electrolyte access and mechanical robustness through cycling. On the other hand, the Zn//2M ZnSO_4_//CL@CTAB_850°C device exhibited 64% capacitance retention after 10,000 cycles, with noticeable fluctuation throughout the cycling performance. This degradation suggests that CTAB alone, used as a soft-templating agent, is insufficient for achieving structural stability and durable electrochemical performance. The absence of chemical activation likely contributes to the limited graphitization, reduced porosity, and poor ion transport, resulting in rapid capacity fading under prolonged cycling. The Zn//2M ZnSO_4_//CL@CTAB@H_3_PO_4__850°C device exhibits a remarkably high initial capacitance retention at early cycles, which can be attributed to the synergetic effect of dual activation. The simultaneous use of CTAB (a soft template) and phosphoric acid (a chemical activator) likely generates an interconnected porous structure with a large accessible surface area, facilitating improved ion diffusion and charge accumulation during early cycles. However, with the continuous cycling, the capacitance retention progressively reduced to 40%, likely due to the structural deterioration and collapse of unstable micropores. These observations highlight that phosphoric acid activation is pivotal in enhancing long-term cycling stability, while CTAB alone does not provide adequate structural durability.

Figure 7C depicts the Coulombic efficiency of all three ZIHSC devices for 10,000 cycles, reflecting the charge-discharge reversibility. The cathode CL@H_3_PO_4__850°C achieves the coulombic efficiency of ∼98% and CL@CTAB@H_3_PO_4__850°C achieves the ∼97% coulombic efficiency, while the cathode CL@CTAB@_850°C achieves the coulombic efficiency of 99%. The coulombic efficiency of the ZIHSCs exhibits noticeable fluctuations over the cycling period. This instability can be attributed to several intrinsic and extrinsic factors. Firstly, the side reactions, such as hydrogen evolution, water decomposition, and electrolyte degradation, are common in zinc-based systems and contribute to the irreversible charge loss. Secondly, initial electrode activation and electrolyte wetting effect may lead to a transient imbalance in charg-discharge behavior, particularly during the early cycles. Thirdly, the non-uniform zinc plating/stripping at the anode surface can promote the formation of zinc dendrites, compromise electrical contact, and isolate the active materials. Finally, the minor leakage current and gradual electrolyte decomposition under prolonged cycling conditions may also contribute to charge insufficiency. These factors collectively result in observed Coulombic efficiency fluctuations, highlighting the need for further optimization of electrode, electrolyte stability, and zinc anode design to ensure consistent cycling behavior.^7^

Self-discharge behavior was studied using the constant current method to assess the practical applicability of the fabricated ZIHSC, as shown in Figure 7D. Devices were initially charged at a 0.1 C rate, followed by a resting period of approximately 3 days under open-circuit conditions. All devices demonstrated excellent voltage retention, indicative of low self-discharge rates.^41^ Specifically, the Zn//2M ZnSO_4_//CL@CTAB_850°C device showed a voltage drop (VD) of 0.43 V, Zn//2M ZnSO_4_//CL@H_3_PO_4__850°C displayed a VD of 0.35 V, and the Zn//2M ZnSO_4_//CL@CTAB@H_3_PO_4__850°C device displayed the VD of 0.4 V. These results translate to voltage retention levels of 72%, 80.5%, and 77.7%, respectively, underscoring the effective retention of stored charge over extended periods without significant loss. This low-self-discharge characteristic is particularly valuable for real-world applications, where energy conversion during device standby is critical. In conclusion, successfully integrating biomass-derived carbon as cathode materials has resulted in high-performance ZIHSC devices with promising electrochemical and self-discharge properties.

### Conclusion

In this work, a self-assembled, templated pulp of *Citrus limetta* was employed as a biomass precursor and subsequently activated using phosphoric acid (H_3_PO_4_) to synthesize porous template biochar, offering a novel biomass-derived cathode material for ZIHSCs. The proposed synthesis strategy is scalable, cost effective, and environmentally benign, presenting a suitable pathway for developing high-performance energy storage systems. The intrinsic properties of the pulp, particularly its fibrous structure, facilitated the formation of a naturally templated architecture, yielding a highly porous and conductive carbon framework with superior electrochemical characteristics. The synergetic effects of biochar’s inherent hierarchical porosity and physicochemical modifications introduced by H_3_PO_4_ activation led to increased surface area, the incorporation of oxygenated functional groups, and optimized PSD. These structural enhancements enabled rapid electrolyte ion diffusion, improved redox surface interactions, and efficient Zn^+2^ ion accommodation, all of which were crucial for advancing the performance of ZIHSCs. Electrochemical analysis of the CL@CTAB@H_3_PO_4__850°C cathode, derived from *Citrus limetta* pulp, demonstrated outstanding performance parameters in aqueous ZIHSCs and showed excellent Coulombic efficiency and self-discharge behavior. This study demonstrates the practical viability of transforming agricultural waste into high-value carbonaceous materials and significantly contributes to energy storage applications. By reducing reliance on non-renewable resources and simultaneously addressing issues related to biomass disposal, the research advances the development of low-cost and resource-efficient electrode materials for next-generation supercapacitor materials.

### Limitations of the study

The present study demonstrated the potential of *Citrus limetta*-derived biochar, prepared through CTAB-assisted hydrothermal treatment, pyrolysis at 850°C, and H_3_PO_4_ activation, for high-performance ZIHSCs. The material demonstrated the stable capacity retention over the 10,000 GCD cycles, indicating promising durability. However, the work does not address how performance might change under the variable real-world conditions, such as fluctuating temperatures and long-term outdoor exposures. In addition, the current synthesis route was tested only on a laboratory scale, and scaling up may represent the challenges related to cost, process control, and consistency in material properties.

## Resource availability

### Lead contact

Further information and requests for resources and reagents should be directed to and fulfilled by the lead contact, Dr. Himmat S. Kushwah (email: himmat.kushwah@jaipur.manipal.edu).

### Materials availability

This study did not generate unique materials.

### Data and code availability

All data supporting the findings of this study are available within the paper and its supplemental information. Additional raw data are available from the lead contact upon reasonable request.

## Acknowledgments

The authors (H.S.K. and M.G.) would like to acknowledge the Manipal University, Jaipur, India, for the Seed Grant Support under the Manipal Research Board Scheme. Also to the 10.13039/501100001852Scientifc Council of CEFIPRA (7105-1) for funding this research.

## Author contributions

M.G.: methodology, conceptualization, investigation, data curation, visualization, and writing – original draft; T.P.: visualization, validation, and formal analysis; R.V.: formal analysis and conceptualization; K.S.: validation, supervision, visualization, and writing – review and editing; H.S.K.: methodology, conceptualization, investigation, supervision, visualization, and writing – review and editing.

## Declaration of interests

The authors declare no competing interests.

## STAR★Methods

### Key resources table

**Table:** 

REAGENT or RESOURCE	SOURCE	IDENTIFIER
Zinc foil (30 cm × 0.25 mm)	Thermo Fisher Scientific	CAS 7440-66-6
Zinc sulfate heptahydrate (ZnSO_4_·7H_2_O, 99 % purity)	Thermo Fisher Scientific	CAS 7440-20-0
Whatman filter paper (125 mm diameter)	Thermo Fisher Scientific	CAS-9004-34-6
Poly (vinylidene fluoride) [PVDF, (-CH_2_CF_2_-)n, M.P. 155-160˚C]	Thermo Fisher Scientific	CAS 24937-79-9
Ethanol (99.8% CH_3_CH_2_OH)	Sigma-Aldrich	CAS-64-17-5
Phosphoric acid (85 wt.% and 99.99% assay, analytical grade)	Rankem Chemical, India	CAS 7664-38-2

### Method details

#### Synthesis procedure of the materials

Citrus limetta pulp was procured from a local juice vendor within the premises of Malaviya National Institute of Technology (MNIT), Jaipur, and thoroughly washed repeatedly with deionized water to eliminate dust and surface impurities, and subsequently dried in a hot air oven (120°C) for 48 hours. The dried biomass was pulverized into fine powder with a mortar and pestle. To synthesize biochar (designated CL@Biochar_350°C), the dried pulp powder was subjected to pyrolysis through a muffle furnace at 350°C for 1.5 hours.

Separately, 6 g of cetyltrimethylammonium bromide (CTAB) was dissolved in 80 mL of DI water with 30 minutes of stirring at 50°C. The obtained CL@Biochar_350°C powder (12 g) was mixed with the prepared CTAB aqueous solution and stirred for half an hour. The resulting mixture underwent ultrasonication for 30 minutes to promote the homogenous dispersion. The prepared solution was sealed in a Teflon-lined stainless-steel autoclave and maintained at 160°C for 6 h for hydrothermal reaction. Once cooled naturally to ambient temperature, the black precipitate was collected, rinsed with deionized water, and dried overnight at 80 °C. Subsequently, the dried material underwent pyrolysis (850 °C) in a muffle furnace for 1 hour. The resultant carbonaceous material was collected and designated as CL@CTAB_850°C.

The CL@CTAB_850°C material was chemically activated using phosphoric acid (H_3_PO_4_) to enhance porosity and surface area. A 1:1 weight ratio of CL@CTAB_850°C was prepared in 50 mL of 0.4M aqueous phosphoric acid solution. The prepared mixture was soaked for 24 hours, followed by filtration using filter paper. The collected precipitate was vacuum dried (80°C) overnight and followed by pyrolysis (850°C) in a muffle furnace for 1 hour. The final solid product was collected and denoted as CL@CTAB@H_3_PO_4__850°C. A parallel activation was performed directly on a CL@Biochar_350°C using a 1:3 weight ratio of biochar to activator (H_3_PO_4_) for comparative analysis. The mixture underwent the same drying and activation conditions as above, and the resulting material was designated CL@H_3_PO_4__850°C. Figure 2 displays a synthesis process of CL@CTAB@H_3_PO_4__850°C material.

#### Fabrication of ZIHSC devices

Cathode slurries were formulated by incorporating 80 wt. % of activated carbon materials (CL@CTAB_850 C, CL@H_3_PO_4__850°C and CL@CTAB@H_3_PO_4__850°C), 10 wt. % PVDF binder and 10 wt. % Vulcan carbon (conductive additive) in ethanol as a dispersing solvent. A homogenous slurry was produced by sonication of the mixture for one hour. The resulting slurries were dropped onto the stainless-steel substrate of a two-electrode Swagelok cell, which served as a current collector. The coated substrates act as cathodes and are vacuum dried for 12 hours at 80°C. The mass loading on the substrate was 1-2 mg cm^-2^.

Zinc-ion hybrid supercapacitor devices were assembled using the prepared carbon-based cathodes, zinc foil as an anode, 2M ZnSO_4_ aqueous solution for electrolyte, and Whatman filter paper for separator. The assembled full cell configuration was designated as follows:

• Zn // 2M ZnSO_4_ // CL@CATB_850°C
• Zn // 2M ZnSO_4_ // CL@H_3_PO_4__850°C
• Zn // 2M ZnSO_4_ // CL@CTAB@H_3_PO_4__850°C

#### Characterization of the prepared samples

Raman spectra of CL@Biochar_350°C, CL@CTAB_850°C, CL@H_3_PO_4__850°C, and CL@CTAB@H_3_PO_4__850°C materials were collected using a 785 nm excitation laser operating at 40.5 mW (PS785 Tracer). Each sample was scanned for 10 seconds under identical acquisition parameters. BET isotherms were determined using a NOVA Touch LX2 gas sorption analyzer (Quanta Chrome Instruments). Before N_2_ adsorption measurements at 77 K, samples were degassed at 200 °C for 6 hours. Functional groups were identified by FTIR spectroscopy in KBr pellet mode (Shimadzu, Kyoto, Japan), scanning over the range of 4000-500 cm^-1^. The morphological characteristics were examined using the Field Emission Scanning Electron Microscope (FE-SEM, Nova-Nano 450, FEI), which offers a resolution of 1.6 nm at 1 kV and less than 1 nm at 15 kV under the TLD-SE mode. The electrochemical performance of fabricated zinc-ion hybrid supercapacitor devices was analysed using the K-Lyte electrochemical workstation. A galvanostatic charge-discharge (GCD), capacity retention, and self-discharge behaviours were analysed by a Battery Testing System (BTS-NEWARE) instrument. The electrochemical impedance spectroscopy (EIS) was evaluated over the range of 0.1 Hz and 100 kHz through a Biologic electrochemical workstation.

### Quantification and statistical analysis

This study does not include a statistical comparison or hypothesis testing. The experimental work focuses on synthesizing and characterizing biomass-derived cathode materials for zinc-ion hybrid supercapacitor devices. All performance metrics and characterization results (e.g., specific capacitance, BET surface area, impedance values, Raman and FTIR spectra) were obtained using the calibrated instruments. No repeated trials were conducted; therefore, values presented reflect individual experiment outcomes without standard deviation and error analysis. Figures were generated and plotted using Origin Pro 2024b Software to ensure clarity in data presentation. All relevant measurement conditions and results were described in the main text, figure legends, and method section.
