# Supplementary material for: Self-assembled templated pulp of Citrus limetta porous Biochar@H3PO4 activated cathode for zinc-ion hybrid supercapacitors
Source: iScience. 2025 Aug 16;28(9):113379. doi: 10.1016/j.isci.2025.113379 (PMC12441688; doi:10.1016/j.isci.2025.113379)
Supplement: Document S1. Figures S1 and S2 and Tables S1–S4 [file mmc1.pdf]

iScience, Volume *28*

Supplemental information

Self-assembled templated pulp of *Citrus limetta* porous Biochar@H_3PO_4 activated cathode for zinc-ion hybrid supercapacitors

Manisha Gautam, Tarun Patodia, Rahul Vaish, Kanupriya Sachdev, and Himmat S. Kushwah

Information for the Supplemental Materials

List of Supplemental Tables:

Table S1: Specific capacitance concerning scan rates

Table S2: Specific capacitance concerning current densities

Table S3: Energy densities Vs. Power densities

Table S4: Comparison analysis of ZIHSC devices with previously reported biomass-based cathode materials for ZIHSCs

List of Supplemental Figures:

Figure S1. Kinetic analysis of ZIHSC devices. (A) b value and (B-D) contribution analysis for**:** Zn // 2M $ZnSO_4$ // CL@CTAB_850°C, Zn // 2M $ZnSO_4$ // CL@H_3PO_4_850° C, and Zn // 2M $ZnSO_4$ // CL@CTAB@H_3PO_4_850°C ZIHSC devices.

Figure S2. Specific capacity Vs. Voltage analysis for ZIHSC devices. (A) Zn // 2M $ZnSO_4$ // CL@CTAB_850° C, (B) Zn // 2M $ZnSO_4$ // CL@H_3PO_4_850° C, (C) Zn // 2M $ZnSO_4$ // CL@CTAB@H_3PO_4_850°C, and (D) Comparison analysis of specific capacity vs. voltage for ZIHSC devices at 0.1 A g^{-1} current density.

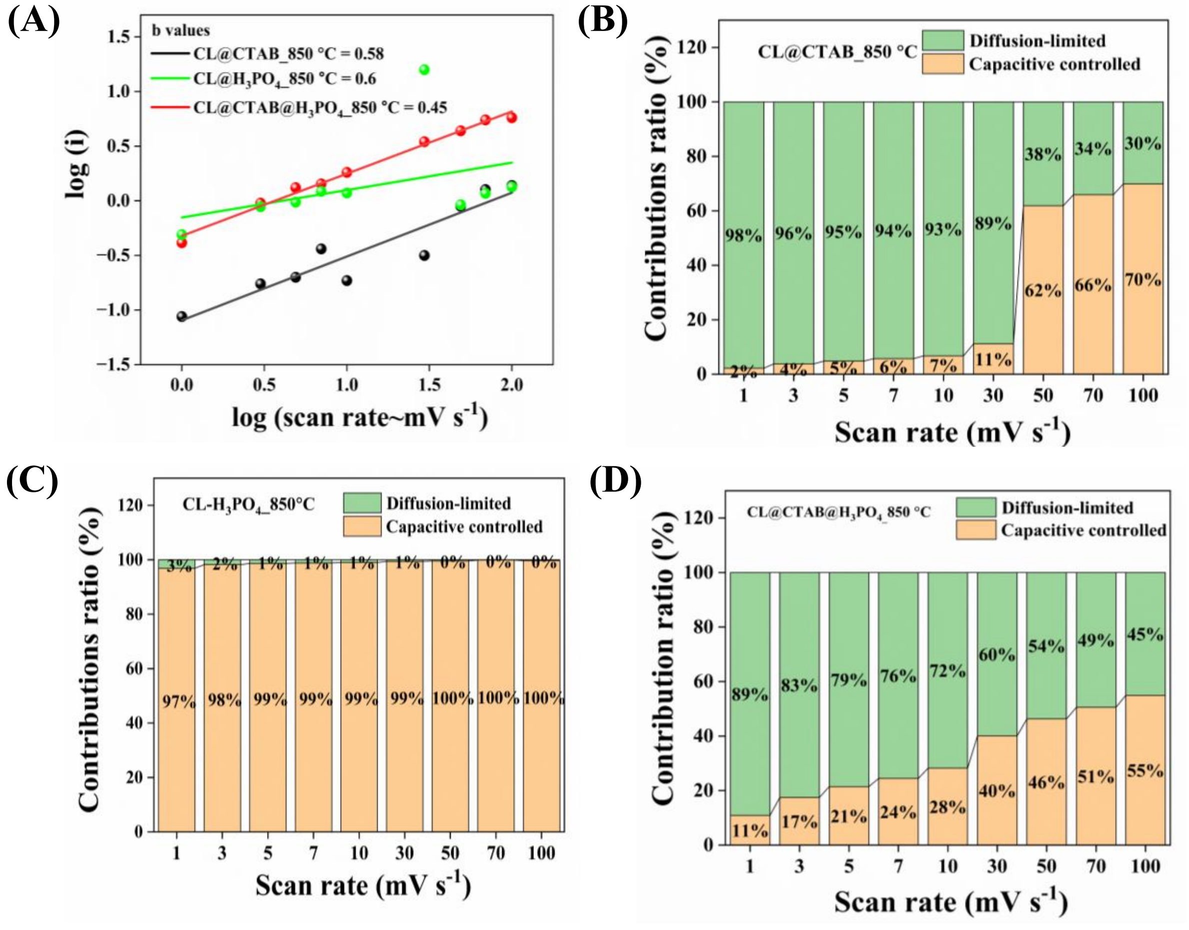

Figure S1. Kinetic analysis of ZIHSC devices. (A) b value and (B-D) contribution analysis for**:** Zn // 2M $ZnSO_4$ // CL@CTAB_850°C, Zn // 2M $ZnSO_4$ // CL@H_3PO_4_850°C, and Zn // 2M $ZnSO_4$ // CL@CTAB@H_3PO_4_850°C ZIHSC devices.

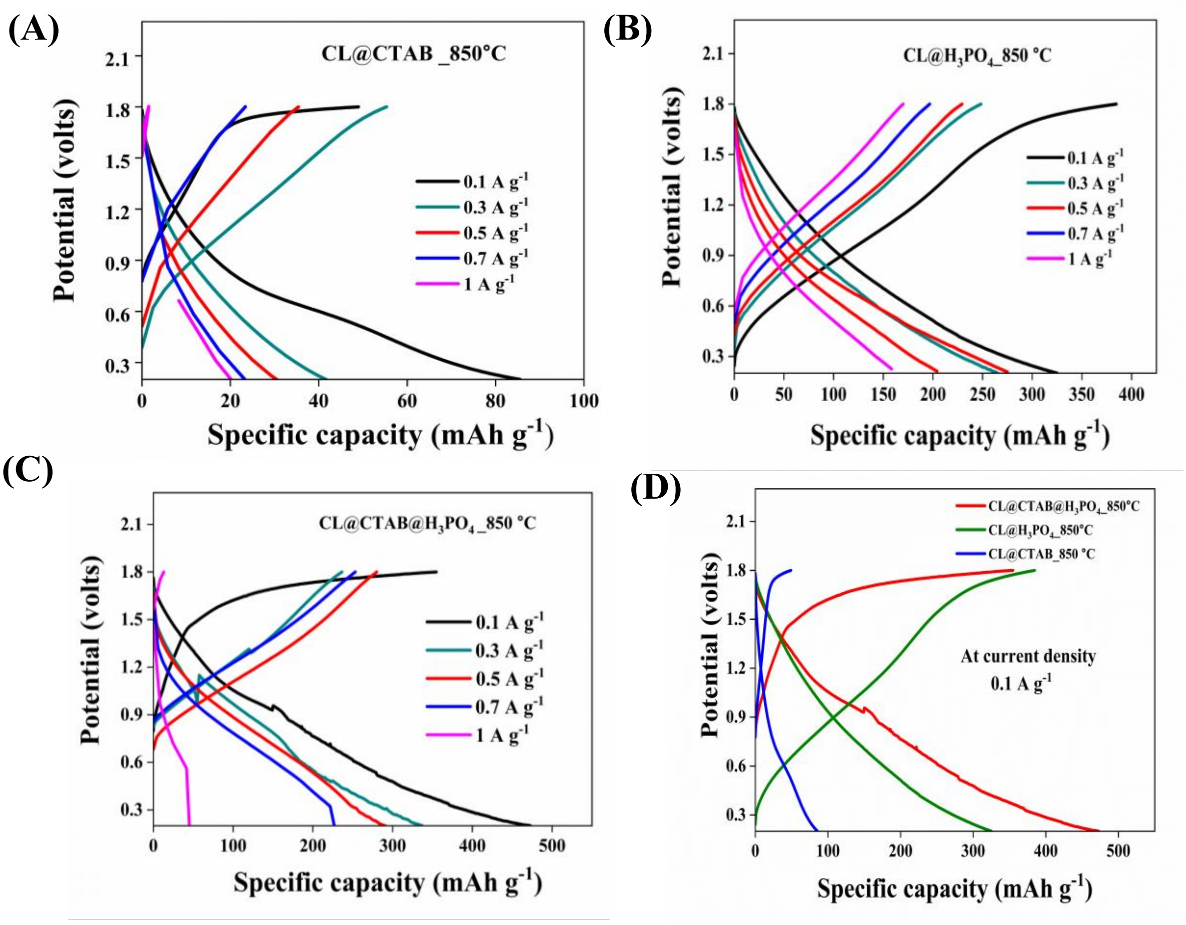

Figure S2. Specific capacity Vs. Voltage analysis for ZIHSC devices. (A) Zn // 2M $ZnSO_4$ // CL@CTAB_850∘C, (B) Zn // 2M $ZnSO_4$ // CL@H_3PO_4_850∘C, (C) Zn // 2M $ZnSO_4$ // CL@CTAB@H_3PO_4_850∘C, and (D) Comparison analysis of specific capacity vs. voltage for ZIHSC devices at 0.1 A g^{-1} current density.

2. Tables:

Table S1: Specific capacitance concerning scan rates

Scan rate (mV s^{-1})	Specific capacitance (F g$^{-1)}$		
	CL@CTAB_850 °C	CL@H_3PO_4_850 °C	CL@CTAB@H_3PO_4_850 °C
**1**	129.51	685.08	693.50
**3**	83.69	485.9	220.51
**5**	57.87	395	317
**7**	72.63	332	230
**10**	51.87	255	150

Table S2: Specific capacitance concerning current densities

Current density (A g^{-1})	Specific capacitance (F g$^{-1)}$		
	CL@CTAB_850 °C	CL@H_3PO_4_850 °C	CL@CTAB@H_3PO_4_850 °C
**0.1**	154.74	697	904.68
**0.3**	98.98	556.44	755.6
**0.5**	79.77	569.45	647.82
**0.7**	101.66	422.82	521.64
**1**	85.56	345	143.7

Table S3: Energy densities Vs. Power densities

Current density (A g^{-1})	Specific capacitance (F g^{-1})					
	CL@CTAB_850 °C		CL@H_3PO_4_850 °C		CL@CTAB@H_3PO_4_850 °C	
	Energy density (Wh Kg^{-1})	Power density (W Kg^{-1})	Energy density (Wh Kg^{-1})	Power density (W Kg^{-1})	Energy density (Wh Kg^{-1})	Power density (W Kg^{-1})
**0.1**	55.019	99	247.82	100	321.6	100
**0.3**	35.192	299.99	197.48	300	268.65	300
**0.5**	28.34	499.56	202.47	500	230.33	500
**0.7**	36.14	699.84	150.33	699.99	185.33	700
**1**	30.42	999	122.46	999.99	51.11	1000

Table S4: Comparison analysis of ZIHSC devices with previously reported biomass-based cathode materials for ZIHSCs

Cathode Materials	Electrolyte	Specific capacitance (F g-1)	Energy density/Power density	Retention rate %	References
Old rice-based porous carbon	2M $ZnSO_4$	164 mAh g^{-1} (0.2 A g^{-1})	131.2 Wh Kg^{-1} (160.1 W Kg^{-1})	10,000 Cycles 94.3 %	[1]
Bamboo fibers	2M $ZnSO_4$	109 mAh g^{-1}	60 Wh Kg^{-1} (1653 W Kg^{-1})	_	[2]
3D Graphene Aerogels	1.5M $ZnSO_4$	353.1 F g-1 (0.1 A g^{-1})	158.9 Wh Kg^{-1} (84 W Kg^{-1}) 14.8 kW Kg^{-1} (77.2 Wh Kg^{-1})	10,000 Cycles 84.2 % At 10 A g^{-1}	[3]
Solanum melongena	2M $ZnSO_4.7H_2O$	313.08 F g^{-1}	141.35 Wh Kg^{-1} (6935.38 W Kg^{-1})	20,000 cycles 98.08 % At 4 A g^{-1}	[4]
Gelatin-based gel biomass	1M Zn $(CF3SO3)_2$	337.6 F g^{-1} 0.5 A g^{-1}	120.1 Wh Kg^{-1} 450 W Kg^{-1}	10,000 Cycles At 10 A g^{-1}	[5]
Gelatin hydrogel	1.5M $ZnSO_4$	176 F g^{-1}	90 Wh Kg^{-1} (445 W Kg^{-1}) 54 Wh Kg^{-1} (14650 W Kg^{-1})	10,000 Cycles 92 % At 5 A g^{-1}	[6]
Groundnut shell	2M $ZnSO_4$	149.5 F g-1 (0.1 A g^{-1})	50.28 Wh Kg^{-1}	2000 Cycles 66 % At 1 A g^{-1}	[7]
Bougainvillea leaves	2M $ZnSO_4$	206.6 F g^{-1} (3 mV s^{-1})	104.8 Wh Kg^{-1} (100 W Kg^{-1})	1200 Cycles 70 % At 1 A g^{-1}	[8]
Banana peels	2M $ZnSO_4$	227.84 F g^{-1} (1 mV s^{-1})	120 Wh Kg^{-1} (5501 W Kg^{-1})	50,000 Cycles 83 % At 1 A g^{-1}	[9]
Citrus limetta	2M $ZnSO_4$	129.51 F g^{-1}	55.01 Wh Kg^{-1}	10,000 Cycles	**This work**

(soft templated porous carbon)		(1 mV s^{-1}) 154.74 (0.1 A g^{-1})	(999 W Kg^{-1})	% At 1 A g^{-1}	
Citrus limetta (H_3PO_4 activated)	2M $ZnSO_4$	685 F g^{-1} (1 mV s^{-1}) 697 F g^{-1} (0.1 A g^{-1})	247.82 Wh Kg-1 (999 W Kg^{-1})	10,000 Cycles At 1 A g^{-1}	**This work**
Citrus limetta (soft templating + H_3PO_4 activation)	2M $ZnSO_4$	69.3.57 F g^{-1} (1 mV s^{-1}) 904.68 F g^{-1} (0.1 A g^{-1})	321.66 Wh Kg^{-1} (1000 W Kg^{-1})	10,000 Cycles At 1 A g^{-1}	**This work**

References

S1. Song B, Xue T, Su R, et al. Puffing-assisted preparation of nitrogen-doped porous biomass carbon for zinc-ion hybrid supercapacitors. *J Anal Appl Pyrolysis*. 2025;186:106906. doi:10.1016/j.jaap.2024.106906

S2. Chen H, Zheng Y, Zhu X, et al. Bamboo-derived porous carbons for Zn-ion hybrid supercapacitors. *Mater Res Bull*. 2021;139:111281. doi:10.1016/j.materresbull.2021.111281

S3. Liu Z, Li G, Cui T, Borodin A, Kuhl C, Endres F. A battery-supercapacitor hybrid device composed of metallic zinc, a biodegradable ionic liquid electrolyte, and graphite. *J Solid State Electrochem*. 2018;22(1):91-101. doi:10.1007/s10008-017-3725-x

S4. Samage A, Halakarni M, Yoon H, Sanna Kotrappanavar N. Sustainable conversion of agricultural biomass waste into electrode materials with enhanced energy density for aqueous zinc-ion hybrid capacitors. *Carbon N Y*. 2024;219:118774. doi:10.1016/j.carbon.2023.118774

S5. Hou X, Ren P, Tian W, et al. High-performance Zn-ion hybrid supercapacitors based on biomass-derived hierarchical porous carbon through template-activated bifunctional induced and ice-crystal assisted strategy. *J Power Sources*. 2024;603:234408. doi:10.1016/j.jpowsour.2024.234408

S6. Zhang Y, Xie P, Jiang C, Zou Z. Nitrogen and oxygen co-doped carbon micro-foams

derived from gelatin as high-performance cathode materials of Zn-ion capacitors. *J Energy Storage*. 2023;57:106169. doi:10.1016/j.est.2022.106169

S7. Gautam M, Patodia T, Kushwaha P, Agrawal M, Sachdev K, Kushwaha HS. Evaluation of zinc-ion hybrid super-capacitor based on chemically activated (KOH/H_3PO_4) ground nutshell biochar. *Carbon Trends*. 2024;15:100341. doi:10.1016/j.cartre.2024.100341

S8. Gautam M, Patodia T, Sachdev K, Kushwaha HS. Bougainvillea flower-biochar for zinc-ion hybrid super-capacitor: role of chemical activator. *Biomass Conversion Biorefinery*. Published online April 6, 2024. doi:10.1007/s13399-024-05547-9

S9. Gautam M, Patodia T, Gupta V, Sachdev K, Kushwaha HS. Synthesis of high surface area activated carbon from banana peel biomass for zinc-ion hybrid super-capacitor. *J Energy Storage*. 2024;102:114088. doi:10.1016/j.est.2024.114088
